# The Role of Wearable Devices in the Management of Congenital Heart Disease

**DOI:** 10.3390/jcm15156111

**Published:** 2026-08-06

**Authors:** Inés Martínez-Saludes, Cristina Ruiz-Herguido, Joan Sanchez de Toledo, David Ferri-Rufete, Silvia Montserrat, David Viñas Fernandez, Eduardo Flores-Umanzor, Raquel Luna-López

**Affiliations:** 1 Cardiology Department, Hospital Clínic de Barcelona, 08036 Barcelona, Spain; imartinezsa@clinic.cat (I.M.-S.); smontser@clinic.cat (S.M.); dvinas@clinic.cat (D.V.F.); flores@clinic.cat (E.F.-U.); 2 Interdisciplinary Cardiovascular Research Group, Institut de Recerca Sant Joan de Déu, Santa Rosa 39-57, Esplugues de Llobregat, 08950 Barcelona, Spain; cristina.ruizh@sjd.es (C.R.-H.); joan.sanchez@sjd.es (J.S.d.T.); david.ferri@sjd.es (D.F.-R.); 3 Cardiology Department, Sant Joan de Déu Barcelona Children’s Hospital, Sant Joan de Déu 2, Esplugues de Llobregat, 08950 Barcelona, Spain; 4 Department of Critical Care Medicine, University of Pittsburgh, Pittsburgh, PA 15260, USA; 5 Cardiology Department, Hospital General de Granollers, 08402 Granollers, Spain; 6 School of Medicine, Universitat Internacional de Catalunya, 08017 Barcelona, Spain

**Keywords:** congenital heart disease, wearable, usability, monitoring sudden cardiac death, minutes of moderate-to-vigorous physical activity, telemedicine, personalized medicine

## Abstract

Patients with congenital heart disease (CHD) represent a special healthcare challenge due to their high complexity, which accompanies them throughout all life stages. Consequently, this population faces increased morbidity and mortality rates, often linked to hemodynamic shifts in pulmonary flow or cardiac output. These risks are further compounded by potential arrhythmias and heart failure decompensation, which may lead to the progressive progression toward advanced stages of the disease. Unfortunately, standard outpatient follow-up is often not capable of responding to the continuous monitoring needs presented by these patients and their families. This selective review frames information on the wearable devices that have emerged as a key solution for continuous and remote monitoring. Beyond clinical tracking, research is increasingly focusing on their role in assessing physical activity—a critical determinant of health outcomes in the CHD population. This review examines the existing literature on wearable technology in both pediatric and adult patients while also addressing the current limitations that hinder their integration into routine clinical practice.

## 1. Introduction

In recent years, health technology has undergone a paradigm shift, transitioning from episodic clinical assessments to continuous, data-driven health surveillance. At the forefront of this transformation are wearables, a subset of digital health technology designed for the non-invasive and longitudinal monitoring of physiological parameters. Unlike traditional diagnostic tools, wearables capture data under real-life conditions, accounting for fluctuations in physical activity, emotional stress, and circadian rhythms. This capability facilitates the identification of digital biomarkers, defined as objective, quantifiable physiological data points that can predict clinical deterioration or evaluate therapeutic efficacy. Key monitored cardiac parameters include heart rate, oxygen saturation, and arrhythmia burden [1].

**Figure 1:**
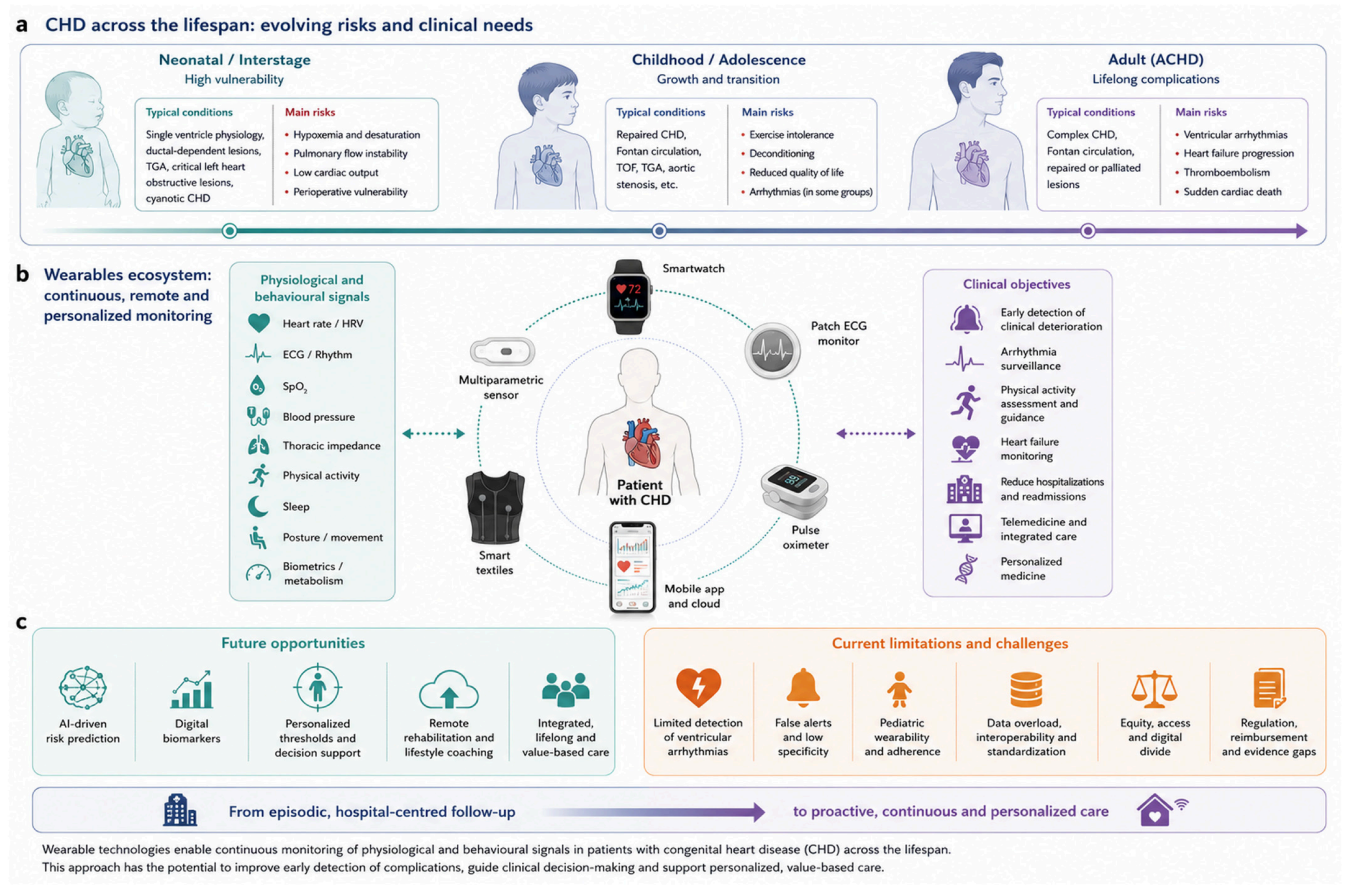
Central Illustration. (**a**) Lifelong trajectory of clinical needs and cardiovascular risks in CHD patients according to age; (**b**) current wearable technologies studied in CHD patients: main monitored parameters, technologies used and clinical outputs; (**c**) future directions.

Patients with congenital heart disease (CHD) represent a population of particular interest due to their high clinical complexity and lifelong care requirements. In early pediatric populations, these devices could prove increasingly valuable for ambulatory perioperative monitoring, potentially providing a critical safety net during high-risk transitions between hospital discharge and home recovery. Furthermore, as survival rates improve, the growing cohort of adults with CHD (ACHD) faces significant long-term morbidity, including heart failure and complex arrhythmias [1]. Wearables offer a transformative shift from reactive to proactive management, potentially enabling the early identification of subclinical deterioration before overt clinical events, informing treatment adjustments, guiding rehabilitation strategies, and empowering patients and families to manage their health. This review summarizes the current status of wearable technology across the CHD lifespan, evaluating its diagnostic accuracy and future trajectories of its integration into standardized clinical workflows. This narrative review was conducted through a structured literature search in PubMed. Searches were performed between December 2025 and April 2026 using combinations of the terms “congenital heart disease”, “adult congenital heart disease”, “Pediatric cardiology”, “wearable devices”, “wearable technology”, “telemonitoring”, “home monitoring”, “physical activity”, and “sudden cardiac death”. Studies published in English were considered. Priority was given to original studies evaluating wearable technologies in pediatric and adult patients with congenital heart disease. Reviews, editorials, and studies not directly assessing wearable-based monitoring in CHD populations were excluded. Final study selection was based on clinical relevance and methodological quality, consistent with the narrative nature of this review. Figure 1 serves as the Central Illustration of the information provided in the manuscript.

## 2. Evolving Epidemiology and Clinical Burden of CHD

With a global prevalence of 9 per 1000 live births, CHD remains a significant clinical challenge [2]. However, thanks to modern therapeutic advances, more than 90% of patients now reach adulthood, including those with the most complex and critical defects [3]. This success has led to a landmark demographic shift where adult patients outnumber pediatric cases [4], increasing the need for sophisticated management that spans the entire patient lifespan [3].

The clinical course of CHD is often marked by complications such as cardiac arrhythmias, heart failure, and thromboembolism, all of which contribute to higher morbidity and hospitalization rates [5]. Although the risk remains a lifelong concern, the specific drivers of mortality vary significantly across age groups. For pediatric patients, the leading causes of death include pulmonary hypoperfusion with critical desaturation events, alongside low cardiac output and heart failure in the immediate post-surgical period [6]. Among these groups, patients with single-ventricle physiology are exceptionally susceptible to these complications. This vulnerability persists from the unrepaired state through palliative Fontan surgery, reaching its peak during the univentricular interstage period. In this phase, dependence on a systemic-to-pulmonary shunt predisposes patients to sudden reductions in pulmonary blood flow and critical desaturation events [5]. Furthermore, a limited capacity to increase systemic cardiac output often leads to hemodynamic instability [7]. Given this clinical fragility, home monitoring programs have already been established, typically centered on tracking oxygen saturation, body weight, and nutritional intake [8]. Similarly, patients with left heart obstructive lesions and cyanotic defects face a significantly elevated risk; conditions such as transposition of the great arteries or aortic stenosis carry a mortality risk 50 to 200 times higher than that of the general population [9].

In contrast, mortality in ACHD is predominantly attributable to heart failure, which remains the leading cause of death, followed by sudden cardiac death (SCD). Both complications are particularly prevalent during the long-term clinical course of patients with Fontan circulation [5]. SCD accounts for approximately 25% of all deaths in the CHD population, with 75% of these cases resulting from ventricular tachycardia or fibrillation [10]. While malignant ventricular arrhythmias affect about 1% of patients over a 10-year follow-up period, this prevalence reaches nearly 10% among those with complex hemodynamic lesions [11]. Consequently, tracking parameters such as blood pressure, heart rate, oxygen saturation, and body weight is paramount, as these have the greatest impact on clinical outcomes in this demographic [5].

## 3. In-Hospital Care Follow-Up vs. Remote Surveillance via Wearables

To address the clinical challenges of CHD, international standards recommend that all patients undergo at least one comprehensive evaluation at a tertiary center to establish an individualized follow-up strategy [2,12]. However, as survival rates and the patient population continue to grow, managing all individuals exclusively within these specialized units has become increasingly unsustainable. Consequently, healthcare models are shifting toward organized networks that incorporate peripheral centers. This framework facilitates a stratified care approach, ensuring that while high-risk patients remain under the direct surveillance of specialized hubs, others can be effectively managed through shared-care protocols with general cardiology services [2,13]. Within these specialized centers, risk assessment focuses on detecting early signs of heart failure, hemodynamic instability, and arrhythmias. In the pediatric population, this necessitates frequent clinical follow-up to closely monitor rapid physiological changes, especially during vulnerable stages or following complex surgical repairs. For adults, risk stratification relies on identifying key predictors of decompensation, including biomarkers of heart failure, assessments of ventricular function supplemented by advanced imaging and periodic Holter monitoring to identify silent but potentially life-threatening rhythmic disturbances [10,11].

Nevertheless, intermittent follow-up by healthcare services presents several limitations. Clinical deterioration in these patients is often rapid and may not be detected early [7]. Information is usually obtained at rest only being representative of part of the daily life clinical situations [1], and it requires frequent visits to healthcare centers, which negatively impact patients’ quality of life [14]. In contrast, home monitoring via wearables integrated with telemedicine could potentially provide a non-invasive and continuous alternative [1] that is significantly less time- and travel-consuming than traditional clinical visits [8]. Such a framework would offer a more comprehensive representation of a patient’s physiological status across diverse real-world scenarios, including periods of physical activity [1]. Its implementation has shown promising preliminary results [7,8], as wearable-based monitoring has been suggested to be associated with the early detection and management of potentially fatal complications [5], reduced number of hospitalizations due to heart failure [8], reduced morbidity and mortality, improved therapeutic adherence, and lower healthcare costs [5].

Finally, remote monitoring addresses the educational and emotional needs of these families. By providing continuous data and a sense of security, these tools facilitate health education and reduce the psychological stress associated with managing such complex conditions [15].

## 4. Physical Activity Monitoring Through Wearables

Beyond physiological surveillance, the objective assessment of physical activity has emerged as a critical research focus, as it serves as a primary determinant of both clinical health and quality of life [15,16,17]. While CHD patients derive significant benefits from regular exercise, the complex interplay of residual anatomical anomalies and vulnerable substrates can place certain individuals at an increased risk of adverse events during physical exertion [18]. In this context, wearables are being investigated for their ability to facilitate safer exercise; they not only allow for the early detection of events during activity but also provide the objective data necessary to prescribe individualized exercise programs more precisely and securely [15,18].

This approach is especially relevant for high-risk cohorts, such as patients with Fontan circulation, whose exercise capacity is often limited by hemodynamic constraints, chronotropic incompetence, and physical deconditioning [19,20]. For these patients, carefully monitored physical activity is vital to mitigate clinical decline. Wearables have shown potential as tools to support home-based cardiac rehabilitation programs, with preliminary studies suggesting feasibility, good adherence, and possible improvements in exercise-related outcomes [15,20].

## 5. Feasibility of Wearable Technology Implementation

While clinical applications of wearables for CHD patients have shown significant promise, their ultimate success hinges on real-world acceptance and wearability, particularly in children and active patients. This concept refers to how effectively a device meets specific user needs while remaining easy to use, making it a decisive factor for long-term clinical integration. Wearability is a multifaceted attribute encompassing physical comfort, practicality, esthetic appeal, and compatibility with a patient’s daily activities [15].

Given the epidemiological profile of the CHD population, there is a high likelihood of widespread acceptance of wearable technologies. Patients and their families are typically young, digitally literate, and often already familiar with mobile health tools. Indeed, many proactively incorporate consumer biosensors into their daily routines, including smartwatches, rings, smart clothing, and biochemical sensors [1]. This predisposition is further supported by the high levels of adherence observed in existing telemonitoring programs; for instance, remote surveillance combined with in-person visits has already become a standard of care for high-risk interstage univentricular patients [1,15].

In the pediatric setting, however, the physical “wearability” of these devices is a paramount consideration. Traditional research-grade tools, such as bulky accelerometers or pedometers, are often technically demanding, expensive, and esthetically unappealing to children, which hinders long-term engagement [18]. To be effective, devices must prioritize a lightweight, non-obstructive design using soft, adjustable materials suitable for different skin and tissue characteristics, including surgical scars.

Furthermore, design requirements must evolve alongside the patient’s developmental stage: infants necessitate strictly non-irritating materials; older children require designs that do not impede mobility; while adolescents and adults prioritize discretion, esthetics, and seamless interactivity [15]. Beyond physical form, technical adaptation remains a challenge; most standard activity trackers are calibrated for adult movement and may fail to capture the brief, intense activity peaks characteristic of childhood, as these devices often register steps or intensity only when the activity exceeds a ten-second threshold [21].

## 6. Essential Wearable Features: Target Populations, Relevant Parameters, and Data Processing

The successful integration of wearable technology into clinical practice hinges on a strategic alignment between device capabilities and the specific hemodynamic and clinical characteristics of patients with CHD. To transform continuous physiological data into clinically meaningful information, three key aspects must be considered: (i) identification of high-risk cohorts most likely to benefit from telemonitoring; (ii) selection of clinically relevant physiological parameters; and (iii) implementation of robust and effective data processing and regulatory strategies. Furthermore, wearable technologies for CHD should be assessed not only as generic consumer devices but also as specialized instruments for risk stratification and personalized rehabilitation. The following section details these essential components and highlights how personalized digital surveillance can address the unique challenges of both pediatric and adult patients with CHD.

### 6.1. Identification of Target Populations and Beneficiaries

Current telemonitoring strategies are primarily focused on patients considered to be at increased risk of heart failure, ventricular arrhythmias, pulmonary blood flow imbalance, and pulmonary hypertension [1,8,22]. Among the populations most frequently studied are patients with Fontan circulation, interstage single-ventricle physiology, complex CHD, frequent healthcare users, and those with limited geographical access to specialized care [5,23]. Additional high-risk groups include patients with Ebstein’s anomaly and repaired Tetralogy of Fallot with risk factors for ventricular arrhythmias [9].

Although current implementation efforts mainly target high-risk populations, wearable technologies may eventually expand to patients with mild-to-moderate lesions that are currently managed conservatively, particularly for the early detection of heart failure progression or arrhythmic complications.

### 6.2. Definition of Clinically Relevant Parameters

Various parameters have been proposed, including heart rate and variability, oxygen saturation, blood pressure, cardiac rhythm and electrocardiogram (ECG), daily step count, impedance and volumetrics, and biomechanical sensors.

Regarding heart arrhythmias and SCD, heart rhythm and ECG are considered critical, as symptomatic arrhythmias require prompt diagnosis and treatment if clinically malignant, and reassurance if benign [5,24]. Nevertheless, most wearables currently feature algorithms specifically designed for the detection of atrial fibrillation through photoplethysmography, single-lead ECG, and mechanocardiography. The ability to detect atrial fibrillation allows for the prevention of thromboembolic events, early rhythm control, arrhythmia progression, and heart failure [5]. Conversely, current wearable devices have a significant limitation in detecting ventricular arrhythmias or other complex arrhythmias, as they lack specialized algorithms for their recognition and are therefore less useful for SCD prevention [25]. Consequently, the use of conventional Holter monitoring and implantable loop recorders is still necessary for monitoring patients at high arrhythmic risk.

Wearable cardioverter-defibrillators (WCDs) deserve specific consideration because they currently represent the only wearable technology capable of both detecting and treating life-threatening ventricular arrhythmias. Although evidence in congenital heart disease remains limited, WCDs may provide temporary protection during periods of transiently increased arrhythmic risk while definitive risk stratification is being completed [26]. In selected patients with ventricular dysfunction, repaired tetralogy of Fallot, a systemic right ventricle, recent cardiac surgery, or newly recognized ventricular arrhythmias, WCDs may serve as a bridge to permanent treatment decisions while avoiding premature ICD implantation. However, further studies are required to better define their role in congenital heart disease-specific sudden cardiac death prevention strategies [27,28].

Concerning heart failure, telemonitoring of weight, blood pressure, heart rate, oxygen saturation, and thoracic impedance, may impact prognosis, as in heart failure in general [25]. Recent devices allow advanced monitoring of parameters such as stroke volume, cardiac output, cardiac index, and systemic vascular resistance, potentially anticipating decompensation in complex patients with chronic hypoxemia and hypotension [5].

### 6.3. Data Management, Regulation, and Processing

Effective data processing is essential for successful telemonitoring. Wearables must provide accurate, actionable alerts that minimize false alarms to prevent patient anxiety and provider fatigue [11,15]. To handle the massive data volumes involved, systems must prioritize the automatic selection of clinically significant information [5]. Furthermore, the lack of formal regulation regarding the transfer of wearable data into patient medical records remains a barrier to informed clinical decision-making [15], representing an especially sensitive challenge in pediatrics.

## 7. Limitations of Wearable Technology Implementation in CHD

CHD patients are difficult to evaluate with standard wearables due to extreme physiological values, heterogeneous disease stages, pediatric representation, and minority status. Consequently, personalized normal ranges are essential to interpret data, avoid false alarms, and detect deviations.

On the other hand, efforts must be made to combat inequity in the adoption of this technology, aiming to make it applicable to as many patients as possible. First, taking into account phenotypic differences such as melanin levels or the size and shape of patients’ body parts is essential. Additionally, ensuring that its use is accessible across different sociocultural environments, with varying levels of familiarity with technology and differing resources, is also noteworthy.

Moreover, as previously mentioned, the lack of algorithms in current commercial wearables for detecting the arrhythmias that most commonly cause SCD, which are validated for atrial fibrillation detection, represents a major limitation in preventing such sudden death, both in patients with CHD and in the general population [25].

Finally, most commercially available wearables are not classified as medical devices, raising unresolved concerns about data reliability, medico-legal responsibility, and the legitimacy of their outputs for guiding therapeutic interventions. Overcoming these challenges will require developing more robust, CHD-specific algorithms and establishing institutional frameworks that explicitly define governance protocols and escalation procedures for wearable-derived alerts within patient care pathways.

## 8. Available Devices Evaluated in CHD

We identified 15 relevant studies analyzing the use of wearables in patients with CHD. These are detailed in the following tables (Table 1 and Table 2). Concerning the representation of the pediatric population, nine of the 15 studies were conducted exclusively in pediatric patients [7,16,17,18,19,21,24,29,30], five focused on adults [6,31,32,33,34] and one of them included both [20]. Within the pediatric cohorts, three studies included patients in their first year of life [7,24,33], whereas the majority focused primarily on patients over 5 years of age [16,17,18,19,20,21,34].

Among the 15 included studies, 8 evaluated health-monitoring devices and 7 focused on physical activity tracking and rehabilitation. Most studies were observational, involved small cohorts, and assessed commercially available wrist-worn devices.

### 8.1. Clinical Evidence for Health Monitoring Wearables in CHD Patients (Table 1)

#### 8.1.1. Arrhythmia Monitoring and Sudden Cardiac Death Prevention

Three studies evaluated wearable devices capable of recording ECGs. They demonstrated generally accurate wave and interval analyses, although P-wave detection proved particularly difficult. This limitation is often exacerbated by the complex atrial anatomy or post-surgical remodeling typical of CHD patients, which complicates rhythm interpretation when compared to the general population. Moreover, most devices are primarily optimized to identify sinus rhythm and atrial fibrillation, often failing to recognize the diverse and complex arrhythmias frequently encountered in CHD. Additionally, rhythm interpretation is frequently hindered by artifacts, and an elevated body mass index, which is common among these patients, presents an additional barrier to high-quality ECG signal acquisition [24,31,32]. Overall, wearable ECG devices demonstrated good agreement with standard ECG measurements, although rhythm classification algorithms showed limited accuracy, particularly in patients with complex congenital anatomy.

Beyond device accuracy, the clinical relevance of rhythm monitoring deserves special consideration in the CHD population. Rhythm surveillance represents one of the most promising applications of wearable technology in patients with congenital heart disease. Given the high prevalence of atrial and ventricular arrhythmias throughout the lifespan of these patients, continuous or repeated rhythm assessment may complement conventional monitoring strategies. However, despite the encouraging accuracy of wearable ECG recordings, current commercial devices remain primarily optimized for atrial fibrillation detection and have limited ability to identify more complex arrhythmias commonly encountered in CHD. Therefore, wearables should currently be considered complementary tools rather than substitutes for conventional rhythm monitoring modalities such as Holter monitoring, event recorders, and implantable loop recorders.

#### 8.1.2. Heart Failure Monitoring

Two studies focused on heart failure parameter monitoring. One of them used a smartwatch and they reported benefits such as shorter hospital stays, reduced healthcare costs, and improved performance in the 6 min walk test (6MWT) [34].

Another study used a custom-built device designed to evaluate cardiac output, showing promising preliminary results when compared to the current gold standard of magnetic resonance imaging [6].

#### 8.1.3. Respiratory Monitoring

Regarding oxygen saturation monitoring, one study evaluating a wrist-worn pulse oximeter found that, while wearables tend to slightly overestimate saturation levels compared to co-oximetry, they may offer higher accuracy than certain standard clinical monitors [33].

Another study involved a wearable patch intended to replace conventional clinical monitors by offering a more convenient, less invasive alternative. This device showed potential for the continuous estimation of heart rate, respiratory rate, and oxygen saturation while also being capable of determining patient posture [7].

#### 8.1.4. Sleep and Quality-of-Life Monitoring

One study used a smartwatch to assess sleep patterns. They have revealed that CHD patients, particularly those with complex lesions, exhibit poorer sleep scores, suggesting that wearables could play a vital role in monitoring the broader impact of the disease on quality of life [33].

### 8.2. Clinical Evidence for Physical Activity Assessment and Rehabilitation Wearables in CHD (Table 2)

The clinical utility of physical activity trackers in the CHD population is heavily dependent on patient adherence and the inherent “wearability” of the devices. Evidence suggests that the degree of required patient engagement is a primary determinant of adherence; specifically, devices requiring active participation to initiate recordings or transmit data [21,32] show significantly lower usage rates than wrist-worn wearables that offer continuous, automated data collection [33]. Regarding form factor, wrist-worn devices generally exhibit superior long-term acceptance compared to waist-worn alternatives [18,19,21], although the latter remain well tolerated for short-term assessments [16]. However, even preferred form factors are susceptible to declining engagement over time due to technical malfunctions, skin irritation, or waning interest in the technology, which may ultimately compromise the reliability of the longitudinal data collected [17,20].

Beyond technical specifications, the socioeconomic and psychosocial context of the patient plays a pivotal role in the success of wearable-based monitoring. Adherence is notably higher when device use is integrated into a framework of close clinical follow-up and social engagement, such as participation in digital social networks [19]. Conversely, a perceived lack of clinical utility, insufficient parental support, or disruptions in the patient’s domestic environment, such as changes in residence, are associated with a marked decrease in compliance [20]. These findings emphasize that the effectiveness of a wearable intervention depends as much on the support ecosystem as it does on the hardware itself.

Furthermore, the anatomical and physiological diversity of CHD patients presents significant challenges for data accuracy, particularly when using commercial devices not tailored to this cohort. Physical characteristics common in CHD, such as central obesity [29], the unique anthropometrics of pediatric patients, and their unpredictable movement patterns, can hinder the correct positioning and function of sensors [7,31]. A critical algorithmic mismatch also exists: most commercial devices utilize adult-validated models optimized for steady-state walking, which tend to systematically underestimate activity in children, whose movement is typically characterized by short, high-intensity bursts [17,21]. Despite these limitations, the measurement of steps and minutes of moderate-to-vigorous physical activity remains largely reliable in CHD patients [21]. Consequently, the integration of these tools into cardiac rehabilitation programs shows significant promise for enhancing and monitoring physical capacity [19,20].

## 9. Future Directions

The clinical implementation of wearables in CHD remains in its early stages, with most applications currently limited to research protocols rather than routine diagnostic use. However, there is growing interest in bridging this gap between exploratory research and daily clinical care. A significant challenge in this transition is the development of algorithms with the high specificity required for critical events such as SCD. While current consumer technology has not yet reached the necessary reliability for independent SCD detection, Artificial Intelligence has already demonstrated preliminary success in specialized settings. This includes predicting acute heart failure [35], identifying cardiorespiratory deterioration in single-ventricle interstage patients [36], and monitoring mortality risk in pediatric intensive care units [37].

Beyond the detection of acute crises, the transition to chronic wearable use could enable the identification of longitudinal physiological patterns that are currently difficult to capture in a clinical setting. Emerging studies suggest that markers such as heart rate variability, arrhythmic burden, peak oxygen consumption, and left ventricular ejection fraction predicted from single-lead ECG signals could serve as non-invasive indicators of cardiac health [1]. For these markers to move from research to clinical practice, significant focus must be placed on improving the ergonomics and “wearability” of devices. Only through high adherence and the collection of continuous data over long periods can these tools provide the reliable trends necessary for proactive patient management.

The future role of activity monitoring is also expected to expand beyond simple data collection. From an equity perspective, wearables could facilitate remote access to exercise and rehabilitation programs for patients who face geographical barriers to tertiary care [19,21]. Furthermore, incorporating these devices into clinical follow-up may provide physicians with a more objective view of a patient’s functional capacity, which has already been shown to positively influence parental perceptions of their child’s quality of life [19]. The engaging nature of these platforms also offers a potential solution to the sedentary behavior frequently observed in this population [20].

Finally, the broader adoption of telemonitoring could contribute to the long-term sustainability of the healthcare system. By reducing the reliance on frequent in-person consultations and minimizing the need for long-distance travel, these tools offer a more efficient model for resource allocation [18]. There is limited emerging evidence suggesting that remote monitoring in CHD patients may be associated with reductions in hospital readmissions, intensive care unit stays, and emergency department visits; however, robust prospective data are still lacking [38]. While widespread clinical integration is still pending, these outcomes suggest that wearables could eventually support a more decentralized and patient-centered care model, optimizing both hospital capacity and patient autonomy.

## 10. Conclusions

The integration of wearables into the management of CHD offers a move from episodic, hospital-based assessments to continuous monitoring in real-life conditions. For pediatric patients, wearable technologies may provide additional support during high-risk periods, such as the interstage phase. In adults with congenital heart disease, they offer a non-invasive approach to continuous physiological monitoring that may help identify early signs of deterioration. However, their ability to improve clinical outcomes remains to be demonstrated through larger prospective studies. Additionally, the objective monitoring of physical activity biomarkers is becoming an essential tool for personalized rehabilitation and improving overall quality of life.

However, for wearables to transition from research-grade instruments to reliable clinical tools, several challenges must be addressed. These include the need for rigorous clinical validation, the development of algorithms specifically tailored to CHD physiology, and the establishment of clear regulations for data management. Despite these barriers, the use of wearable technology represents a necessary step toward a more proactive and personalized care model, better suited to the lifelong complexity of congenital heart disease.

## Figures and Tables

**Table 1:** Clinical studies evaluating health monitoring wearables in CHD.

Study, Year (Ref)	Population	Pathology	Summarized Intervention	Study Variables	Key Results
Borrelli et al., 2025 [34]	25 adults; mean age 38.35 ± 11.33 y	UVH after palliation (*n* = 9); repaired TOF (*n* = 5), SRV (*n* = 4), systemic outflow obstruction (*n* = 4), VHD (*n* = 3)	Telemonitoring using a BioBeat watch (BioBeat Technologies Ltd., Boca Raton, FL, USA) and app with regular clinical review.	6MWT, vital signs, NYHA FC, pro-BNP levels, LVEF, patient satisfaction, and economic indicators.	Reduced hospital days (4 vs. 7; *p* = 0.04) and costs per patient (*p* = 0.02).
Ganti et al., 2022 [6]	45 adults; mean age 15 ± 7.9	Mixed CHD	Custom ECG/SCG wearable to estimate SV compared with cardiac MRI.	SV.	Mean SV error was 28%, falling within the acceptable 30% threshold.
Harris et al., 2019 [29]	22 infants; median age 13 d (2–195 d)	HLHS (*n* = 7); TA (*n* = 1); DORV (*n* = 5); TGA (*n* = 1); PuA (*n* = 1); TAPVR (*n* = 1); TOF/PuA/MAPCAs (*n* = 3); other CHD (*n* = 5)	Comparison of SpO_2_ [WristOx2 (Nonin Inc., Plymouth, MA, USA) and Masimo devices (Masimo Inc., Irvine, CA, USA)] vs. SaO_2_ (co-oximetry).	SpO_2_.	Neither wearable met equivalence criteria with co-oximetry.
Kobel et al., 2022 [24]	215 children; mean age 8.05 ± 4.33 y	Normal heart (*n* = 134); VSD (*n* = 31); ASD (*n* = 16); TOF (*n* = 10); TGA (*n* = 10); HLHS (*n* = 10); HRHS (*n* = 2); Shone complex (*n* = 2)	Apple Watch (Apple Inc., Cupertino, CA, USA) (Lead I equivalent) compared with standard 12-lead ECG.	Heart rhythm, ECG waves, and interval measurements.	Good correlation in all ECG measurements. Automated algorithm failed for rhythm classification in 37.9% of cases, compared with 5.1% for cardiologists
Runge et al., 2025 [32]	9 adults; median age 33 y	TGA (*n* = 4); Fontan circulation (*n* = 1); TOF (*n* = 1); VSD with PuA (*n* = 1); CoA (*n* = 1); uncorrected cyanotic CHD (*n* = 1)	Kardia Mobile recordings (AliveCor Inc., Mountain View, CA, USA) (3 × 30 s iECG/week) compared with expert reviews.	Heart rhythm.	Sensitivity (52%) and specificity (45%) for automated SR detection were low.
Schöneburg et al. 2025 [33]	175 adults; mean age 33.1 ± 10.3 y +52 controls	Simple (*n* = 17); moderate (*n* = 81); complex (*n* = 77)	Continuous 7-day sleep tracking using a Garmin Vivosmart 5 (Garmin Ltd., Olathe, KS, USA)	Sleep quality parameters.	Complex CHD correlated with poorer sleep scores compared to simple or moderate CHD.
Striepe et al., 2022 [31]	106 adults; mean age 34 ± 13.22 y	TOF (*n* = 24); structurally normal heart (*n* = 22); AS (*n* = 18); VSD (*n* = 13); UVH (*n* = 10); ASD (*n* = 8); TGA (*n* = 9); TAC (*n* = 2)	Apple Watch 4 (Apple Inc., Cupertino, CA, USA) (Leads I, II, and III equivalents) compared with standard 12-lead ECG.	Heart rhythm, ECG waves, and interval measurements.	77.4% rhythm agreement; Lead III was uninterpretable in 12 cases due to central obesity.
Zhou et al., 2025 [7]	19 infants;	Single-ventricle physiology	Custom chest and forehead patches compared with standard hospital ICU monitors.	HR, RR, skin temperature, body posture, and SpO_2_.	Strong HR agreement (R^2^ = 0.99); forehead placement reduced SpO_2_ motion artifacts.

Abbreviations: y: years, *n*: number of patients, UVH: Univentricular Heart, TOF: Tetralogy of Fallot, SRV: Systemic Right Ventricle, VHD: Valvular Heart Disease, HF: Heart Failure, 6MWT: Six-Minute Walk Test, NYHA FC: New York Heart Association Functional Class, pro-BNP: Pro-brain Natriuretic Peptide, LVEF: Left Ventricular Ejection Fraction, CHD: Congenital Heart Disease, ECG: Electrocardiogram, SCG: Seismocardiogram, SV: Stroke Volume, MRI: Magnetic Resonance Imaging, d: Days, HLHS: Hypoplastic Left Heart Syndrome, TA: Tricuspid Atresia, DORV: Double Outlet Right Ventricle, TGA: Transposition of the Great Arteries, PuA: Pulmonary Atresia, TAPVR: Total Anomalous Pulmonary Venous Return, MAPCAs: Major Aortopulmonary Collateral Arteries, SpO_2_: Oxygen Saturation, SaO_2_: Arterial Oxygen Saturation, VSD: Ventricular Septal Defect, ASD: Atrial Septal Defect, HRHS: Hypoplastic Right Heart Syndrome, CoA: Coarctation of the Aorta, iECG: Mobile Electrocardiogram, SR: Sinus Rhythm, AS: Aortic Stenosis, TAC: Truncus Arteriosus Communis, ICU: Intensive Care Unit, HR: Heart Rate, RR: Respiratory Rate, R^2^: Coefficient of Determination.

**Table 2:** Clinical studies evaluating activity tracking wearables in CHD.

Study, Year (Ref)	Population	Pathology	Summarized Intervention	Study Variables	Key Results
Brudy et al., 2020 [18]	162 children; mean age 11.8 ± 3.2 y	96 controls. LHO (*n* = 28), RHO (*n* = 53), isolated shunts (*n* = 20), TGA post-switch (*n* = 19), total cavopulmonary connection (*n* = 25), miscellaneous (*n* = 17)	7-day monitoring using a Garmin vivofit Jr (Garmin Ltd., Olathe, KS, USA)	Steps, minutes of MVPA, and subjective PA	75.9% met WHO recommendations *. Severe CHD associated with not reaching targets.
Brudy et al., 2021 [30]	343 children; mean age 12.1 ± 3.3 y	Complex (*n* = 162); moderate (*n* = 109); simple (*n* = 64); 8 others	7-day monitoring with a wrist-worn Garmin vivofit Jr. (Garmin Ltd., Olathe, KS, USA)	Daily steps, daily minutes of MVPA, quality of life	78% met WHO recommendations *. MVPA associated with HRQoL.
Fernie et al., 2023 [20]	26 patients; mean age 13.5 ± 3 y	Single-ventricle physiology	12-month exercise program monitored with Garmin Vivosmart 4 (Garmin Ltd., Olathe, KS, USA)	CPET data, 6MWT data, and self-efficacy	Reduced resting HR (*p* = 0.04) and increased pre–post SBP (*p* = 0.004) and minute ventilation (*p* = 0.012) at peak exercise.
Jacobsen et al., 2016 [19]	13 children; median age 10 (8–12) y	Fontan	12-week rehab program monitored via Fitbit Flex (Fitbit Inc., San Francisco, CA, USA) and daily reports	PedsQL, Shuttle Run results, and activity data	Significant improvement in VO_2_max (*p* = 0.001) and parent-reported HRQoL (*p* = 0.004).
Kuan et al., 2020 [17]	156 children; mean age 12.7 ± 2.4 y	TOF (*n* = 38); CoA (*n* = 47); TGA (*n* = 29); Fontan circulation (*n* = 42)	12-month monitoring with Fitbit Charge 2 (Fitbit Inc., San Francisco, CA, USA); 1-week validation with ActiGraph (Actigraph LLC., Pensacola, FL, USA)	Daily step count, types of PA, and quality of life	Adherence declined to 60% due to loss of interest, technical issues, and skin irritation.
Voss et al., 2017 [16]	90 children; mean age 13.6 ± 2.7 y	Mild (*n* = 26), moderate (*n* = 26), severe (*n* = 29)	7-day monitoring with hip-worn Actigraph (Actigraph LLC., Pensacola, FL, USA)	PA, quality of life, and climate parameters	Only 8% met WHO recommendations *.
Voss et al., 2017 [21]	30 children; mean age 13 ± 2.2 y	CHD (*n* = 24); transplant (*n* = 1); EP disorder (*n* = 1); controls (*n* = 3)	Concurrent 7-day monitoring using Fitbit Charge HR (Fitbit Inc., San Francisco, FL, USA) and waist-worn Actigraph (Actigraph LLC., Pensacola, FL, USA)	Step count, minutes of MVPA, and sedentary time	Strong step agreement (ICC = 0.8855). Only 25% achieved WHO recommendations *.

Abbreviations: y: years, *n*: number of patients per group, LHO: Left Heart Obstruction, RHO: Right Heart Obstruction, TGA: Transposition of the Great Arteries, MVPA: Moderate-to-Vigorous Physical Activity, PA: Physical Activity, WHO: World Health Organization, CHD: Congenital Heart Disease, HRQoL: Health-Related Quality of Life, CPET: Cardiopulmonary Exercise Testing, 6MWT: Six-Minute Walk Test, HR: Heart Rate, SBP: Systolic Blood Pressure, PedsQL: Pediatric Quality of Life Inventory, VO_2_max: Maximum Oxygen Consumption, TOF: Tetralogy of Fallot, CoA: Coarctation of the Aorta, EP: Electrophysiological, ICC: Intraclass Correlation Coefficient. * WHO recommendation of daily PA: 60 min per day.

## Data Availability

No new data were created or analyzed in this study. Data sharing is not applicable to this article.

## Abbreviations

The following abbreviations are used in this manuscript:

ACHD	Adult Congenital Heart Disease
ASD	Atrial Septal Defect
CHD	Congenital Heart Disease
CoA	Coarctation of the Aorta
CPET	Cardiopulmonary Exercise Testing
d	Days
DORV	Double Outlet Right Ventricle
ECG	Electrocardiogram
EP	Electrophysiological
HF	Heart Failure
HLHS	Hypoplastic Left Heart Syndrome
HR	Heart Rate
HRHS	Hypoplastic Right Heart Syndrome
HRQoL	Health-Related Quality of Life
ICC	Intraclass Correlation Coefficient
ICU	Intensive Care Unit
iECG	Mobile Electrocardiogram
LVEF	Left Ventricle Ejection Fraction
MAPCAs	Major Aortopulmonary Collateral Arteries
MRI	Magnetic Resonance Imaging
MVPA	Moderate-to-Vigorous Physical Activity
NYHA FC	New York Heart Association Functional Class
PA	Physical Activity
PedsQL	Pediatric Quality of Life Inventory
Pro-BNP	Pro-brain Natriuretic Peptide
PuA	Pulmonary Atresia
RR	Respiratory Rate
RSV	Systemic Right Ventricle
R^2^	Coefficient of Determination
SaO_2_	Arterial Oxygen Saturation
SBP	Systolic Blood Pressure
SCD	Sudden Cardiac Death
SCG	Seismocardiogram
SpO_2_	Oxygen Saturation
SR	Sinus Rhythm
SV	Stroke Volume
TA	Tricuspid Atresia
TAC	Truncus Arteriosus Communis
TAPVR	Total Anomalous Pulmonary Venous Return
TGA	Transposition of the Great Arteries
TOF	Tetralogy of Fallot
UVH	Univentricular Heart
VHD	Valvular Heart Disease
VO_2_max	Maximum Oxygen Consumption
VSD	Ventricular Septal Defect
WHO	World Health Organization
y	years
6MWT	6-Minute Walk Test
